# Enhancement of Online Education to the Teaching Paradigm: Taking Academic Medical Postgraduate Cultivation as an Example

**DOI:** 10.3389/fmed.2022.807469

**Published:** 2022-03-30

**Authors:** Bin Su

**Affiliations:** ^1^Beijing Key Laboratory for HIV/AIDS Research, Clinical and Research Center for Infectious Diseases, Beijing Youan Hospital, Capital Medical University, Beijing, China; ^2^Sino-French Joint Laboratory for Research on Humoral Immune Response to HIV Infection, Clinical and Research Center for Infectious Diseases, Beijing Youan Hospital, Capital Medical University, Beijing, China

**Keywords:** medical education, postgraduate training, academic medical postgraduates, multi-dimensional teaching mode, online education

## Abstract

**Background:**

The COVID-19 pandemic posed enormous challenges to postgraduate teaching in 2020. Large-scale and continuous online teaching explorations were introduced to cope with this difficult situation, which incidentally shifted the paradigm of postgraduate teaching.

**Purpose:**

A review of the online teaching of local medical schools for postgraduates was performed to identify the success factors in realizing the practice.

**Methods:**

We retrieved medical postgraduate online teaching publications mainly from the local database, the China National Knowledge Infrastructure (CNKI), *via* the keywords stated below and then performed a retrospective analysis.

**Results:**

We analyzed key success factors in improving online learning engagement that were considered exclusive to offline classroom teaching, including emotional interaction, the immediacy of communication, and enthusiasm for participation. With these positive effects, the integration of online and offline teaching advantages is beneficial for the initiative of academic medical postgraduates and promotes the construction and development of medical postgraduate education.

**Conclusion:**

Online education can overcome the limitations of time, space, and teaching frequency, with great advantages in terms of flexibility and mobility over traditional classroom teaching. It can effectively cope with difficulties in the education of academic medical postgraduates in challenging times. In the post-pandemic era, blended online and offline teaching approaches continue and will become the new normal pedagogy for the training of medical postgraduate students.

## Introduction

In recent decades, the rapid development of the Internet and information technology has greatly propelled traditional classroom teaching. Online teaching is widely applied to different majors in various universities, and the use of network technology and resources to enhance teaching activities is increasingly popular among teachers (1–3). Indeed, for the time being, online education is used only as a dispensable aid to traditional classroom teaching (4). It was hardly conceivable that from the beginning of 2020, online education could replace the face-to-face teaching approach in the classroom with such an abrupt transition as a result of the sudden outbreak of the coronavirus disease 2019 (COVID-19) pandemic. However, in response to the Chinese Ministry of Education's policy requirements of “disrupted classes, uninterrupted learning”, online education, which had previously played an auxiliary and experimental role, has swept through China's basic and higher education almost overnight (3, 5–7). Teachers and students suffering through this extraordinary period have undergone changes from being caught off guard and preparing nervously to resisting and silently accepting online teaching and then to gradually groping for a normal and orderly method (8, 9). In the post-pandemic era, students and teachers in most areas have returned to the classroom, yet the impact and transformation of online education has made the classroom no longer exactly what it used to be (10–13). Public health events have made a profound impact on and changed teaching methods in the internet age.

Postgraduate online education was also among the issues of concern during the pandemic. By searching the Web of Science, Liu et al. (14) analyzed 1,243 articles published in August 2021 and found that foreign studies on online education mainly concentrated on compatibility, content requirements, the use of popularization technologies, project design for advanced ubiquitous learning (15), the learning process and randomized controlled trial design. By searching for the keyword “online education” in the China National Knowledge Infrastructure (CNKI, https://www.cnki.net), we found that there were already 7,899 articles reporting local online teaching practices and presenting a variety of trials exploring different online education platforms, modes, systems, and prospects (8, 16–18); furthermore, there have been endless commentaries on the advantages and disadvantages of online education vs. traditional classroom education as well as their relationship (19–22). Those studies have provided a very comprehensive analysis of their strengths and weaknesses. Most scholars have fully affirmed the flexibility of online education in time and space. Online education is characterized by “being able to learn anywhere and at any time”; that is, it is not limited by time, place or the frequency of teaching (23). This teaching approach breaks through the limited resource space and interactive scenes of classroom teaching, shortens the space-time distance of communication, and gives postgraduates more flexibility and independence in learning (24).

In this study, we systematically reviewed online education and offline traditional teaching practices in medical universities for the cultivation of academic medical postgraduates. Through a holistic evaluation, we identified some success factors that are necessary for effectiveness and instrumental metrics for the online teaching approach. Online teaching can benefit postgraduate education despite some of its drawbacks, can be to overcome over time and with the development of technologies. Even in the post-pandemic era, teachers and students have returned to the classroom, and online teaching is increasingly valued. For example, we can adopt hybrid teaching that integrates online and offline approaches to give full play to the strengths of online education for training academic medical postgraduates.

## How to Turn the Disadvantages of Online Teaching into Advantages in the Cultivation of Postgraduates

### 1. The Strengths and Weaknesses of Online Teaching for Medical Postgraduates and the Feasibility of Realizing a “Homogenization” of Teaching Mobility and Quality

Compared to the traditional classroom, online teaching is easier to implement student-centered, personalized learning rather than an indiscriminate teaching consisting of “chalk and talk” (25). However, studies point out that online teaching is subject to student's self-discipline, uncertain learning environments, computer skills, and the network (26, 27). The lack of face-to-face interaction with teachers, the response time and the absence of traditional classroom socialization were also highlighted by higher education students. Some deemed online teaching hardly able to stimulate the enthusiasm of postgraduates to learn due to the lack of group incentives and supervision by teachers, like in the traditional classroom (24). Exploring more effective postgraduate education approaches remains a hot topic investigated both domestically and internationally from different perspectives (28–30). China's postgraduate education has reformed to adopt “dual-track” modes, “academic” and “professional,” based on different cultivation objectives (31, 32). The academic mode aims to cultivate talents with strong medical research capabilities or clinicians with strong research capabilities (33, 34). However, confined by the traditional training of medical postgraduates, the cultivation of academic medical postgraduates still has some shortcomings. Generally, supervisors assign a great deal of clinical work to students. It may improve student's clinical skills and practical experience, although students lack the time to exercise their scientific research ability (35). At the other extreme, students are arranged for onerous scientific research experiments, which subjectively equate “basic experiments” with “scientific research” and lead to a disjunction of clinical practice, theory, and scientific research to some extent (36). Both approaches often take a considerable amount of precious time and therefore hardly provide a guarantee that students have enough time to study theories and carry out classroom discussions. Based on the cultivation objective, academic medical postgraduates should be able to master basic and cutting-edge theories, advanced technology, scientific research, and innovation thinking from the standpoint of their majors. However, in the current situation, academic postgraduates are first required to receive a considerable number of courses and necessary training for experimental skills at first; otherwise, in the absence of theoretical training and experience, most postgraduates do not even have the ability to independently determine their future research projects (37). In this context, with its unique flexibility and mobility, online teaching can compensate for these shortcomings and demonstrate its irreplaceable advancement. Since online teaching is relatively free from time limitations, lectures and academic discussions can be arranged more specifically and better guarantee the students will receive a theoretical and scientific research education. Moreover, the recording and playback function allows students to watch repeatedly based on their own needs after class and meet the individual requirements of students based on their basis and learning abilities. The flexibility of online teaching at any time is thus of particular significance to medical postgraduates.

In addition, medical postgraduates face spatial limitations. For example, confined to the requirements and restrictions of joint training policies, beds, and the number of training programs, medical schools usually assign medical postgraduates to different training bases (38). As a result of such a training arrangement postgraduates, even under the same supervisor, cannot gathering together in one place for study, and traditional classroom teaching cannot be realized. Meanwhile, due to differences in teaching resources and teachers' proficiency between various practice bases, it is difficult to guarantee the “homogenization” of teaching quality (30, 39). To address this issue, the advantage of online education is embodied once again by being free from geographical restrictions and being able to ensure that students in different places receive uniform learning and mentoring. Students can also keep abreast of the progress and level of their classmates, the requirements of supervisors, and the trend of the subject so that they can make adjustments accordingly (40, 41).

From the above analysis, the main strengths of online teaching allow for the flexibility of teaching without time limitations as well as feasibility without spatial limitations. The weaknesses of online teaching mainly lie in the lack of network coverage, computer skills, and measurements for maintaining teaching disciplines. All these facts have also been observed in teaching practice in other countries; however, the mainstream views tend to believe that online teaching will become an integral part of teaching approaches for improving teaching quality, motivating student's interest in learning and reducing learning costs (42, 43).

### 2. Online Teaching Does Not Decrease but Increases the Classroom Participation

Traditional classroom teaching is generally believed to be very convenient for teachers and students to engage in face-to-face communications. Especially in small-class teaching, teachers can easily judge postgraduate's understanding of the lecture content through eye contact, tone of voice, and question-and-answer interaction, and accordingly adjust the teaching progress in real time (44, 45). In contrast, with online teaching, it is harder to maintain such immediate feedback and adjustment. However, our teaching practice and other studies have indicated that postgraduates become even more active in online teaching than in offline traditional teaching. Although there is a lack of real-time eye contact and feedback in offline classrooms, students can leave messages and comments in the teaching moment through auxiliary teaching software such as DingTalk or Tencent (46, 47). Studies further reveal that online teaching can allow for a relaxed atmosphere for on-the-spot comments and discussion. First, in the traditional classroom layout, teachers often stand at the front and face students, leading to an opposing and top-down power-suppressing relationship with students and a nervous atmosphere (48). In contrast, in the online teaching environment, teachers just appear as icons or are projected on a screen, which greatly relieves this tension. Second, in face-to-face teaching, students need to spend more time on psychological preparation to overcome their shyness and stage fright in speaking in front of the class and, at the same time, ensure the integrity of their content and language and accept the teacher's judgment (49). However, leaving a text message is more casual since the message can be long or short, and many students will even use emojis to express their psychological reaction. Although there is a lack of eye contact and oral expression, like in the traditional classroom, students generally think that the use of text messages and emojis is an equally good way of emotional communication in the classroom, particularly those who have the intention to talk but are hesitant to do so. Not only does this method of communication give students a greater sense of security, but it also brings a relaxed atmosphere to the class. In an article aiming to promote the construction of postgraduate courses in the online teaching mode, Mo et al. pointed out that online teaching could increase the interaction between teachers and students. In the process of teaching supervision, we also found that more postgraduates were enthusiastic about speaking in WeChat groups or on conferencing platforms in online teaching than in previous offline classrooms. The reason is that when speaking in front of the class, students are required to organize their language and make eye contact with everyone, which therefore makes students feel intimidated. However, all these factors disappear in online communication. Students become more active in speaking, and teachers can better receive teaching feedback as well (50). The ability of online teaching to enhance teacher-student interaction also appears in scenarios other than postgraduate teaching. For example, in interviews with teachers in elementary and middle schools, teachers generally believe that online interaction is more frequent than in offline interaction. Online communication does not require face-to-face communication, which breaks down the psychological defenses line of some students with poor communication. They can choose to communicate with teachers in private messages online; students do not have to worry about losing face to ask questions that may be laughed at by others (23).

Another noteworthy phenomenon is that when teachers deliver online lectures and postgraduates present academic reports, speakers usually receive more feedback than they would offline. Although feedback is not realized through eye contact and the exchange of expressions, students give immediate feedback by receiving messages and comments in the teaching process. This kind of feedback is more specific and constructive and almost impossible to realize in the traditional classroom. Students can also record their doubts and evaluations in a timely manner by leaving a message without worrying about disturbing lecturing and reporting. The lecturer can not only see the feedback in real time but also choose a suitable time to adjust the lecture content and answer questions more easily. Therefore, online teaching is conducive to creating a relaxed class environment that is able to enhance student's enthusiasm for learning and desire to express themselves, increase student's classroom participation and improve teaching quality.

### 3. Online Teaching Promotes the Realization of “Student-Centered” Learning

Educators have been continuously pursuing effective teaching modes, including the postgraduate education paradigm (7, 13, 34, 51). The traditional classroom has been in existence for hundreds of years. It is a typical class-based teaching paradigm and a stably organized teaching activity. However, it has difficulties in meeting the individualized learning needs of students, and it is not conducive to cultivating student's independent learning ability (52). The flipped classroom has been vigorously promoted in recent years, with the purpose of defending students' learning autonomy with the support of information technology, adjusting the teaching relationship, and providing a basic guarantee for student's independent learning and individualized learning (53). By flipping the teaching process, the flipped classroom turns the golden rule of teaching in traditional classrooms from “pre-class preparation → in class → after-class reviewing” to “preparation → video-watching → application” (54) to realize the flip from “teacher-centered” to “student-centered” teaching modalities. In the post-pandemic era, online education has gradually become popular, and a new mode of blended online and offline teaching has also appeared in postgraduate education. Such blended teaching combines the online and offline modes, fully leveraging the features of the flipped classroom to mobilize student's initiative and autonomy in learning (25). The blended teaching mode of “combining teacher-led practice and student's self-independent learning” not only raises the initiative of learning subjects but also imposes more requirements on teachers. Teachers must not only play the role of traditional supervisors but also fully mobilize the initiative and enthusiasm of students in the learning process. Teachers are required to know not only “how to teach” but also “how to promote learning” (55). This transformation in teaching is particularly important in postgraduate education. Postgraduate education has higher standards for teaching, which should be professional, cutting-edge, and cross-disciplinary. It also requires students to have speculative and innovative thinking. All these requirements become the focus of postgraduate education and a difficulty in postgraduate education, and they result in higher standards for cultivation. High-quality postgraduate education should not be confined to traditional classes for the teaching of basic knowledge or a unilateral requirement for the proficiency of teachers. Postgraduates should be able to acquire the latest progress and skills related to their majors from various channels and obtain learning motivation and inspiration from the feedback of teachers and mutual discussions with their peer classmates. The democratization of online education will help students and teachers fulfill these demands more effectively. With the full development of online teaching, teachers and students can build QQ and WeChat groups to study and discuss the materials issued by teachers and work together on assigned tasks. In addition, they can share the scientific research papers they read and report their progress in their own research fields. Teachers and students can communicate easily in real time, and all learning processes can receive feedback and evaluation. It is through this multifaceted learning and discussion that online teaching promotes the realization of individualized learning, and with a timely feedback mechanism, online teaching further enhances student's self-learning and the transformation of the student-centered teaching mode.

This teaching mode can rule out the questions that students can solve by themselves and screen out those that deserve to be discussed more through in-depth face-to-face communication and in which hypotheses can be proposed to be tested in practice. Through the blended online and offline teaching mode, postgraduate education has changed from the “pre-class preparation, video-watching, and application” adopted in the flipped classroom to a multidimensional mode of “pre-class preparation, class, discussion, feedback, and application.” As a result, teaching interaction has entered an alternating online and offline cycle, creating a rich learning experience. The autonomy of discovering, discussing, and solving problems and a more relaxed learning atmosphere have enabled many students to acquire a deep learning ability. Students can not only learn actively and critically but also have a deeper understanding and improved ability to transform the knowledge they have learned (56). Students enjoy the whole learning process in this mode, which can improve the mutual benefits between teachers and students and update the cutting-edge knowledge and scientific progress among classmates. Additionally, this mode may be beneficial for supervisors to monitor their own teaching effectiveness and the progress of students' projects (12).

## Conclusion

In recent decades, with the development of teaching technology, people have been tirelessly exploring the organizational form of the classroom. These attempts have greatly enriched the forms of teaching and initiated a revolution in teaching. Currently, online education is aggressively entering into the daily teaching and training of postgraduates, which poses not only a challenge for postgraduate education, especially for academic medical students, but also provides a rare opportunity for great change. Offline teaching has hundreds of years of theoretical and practical history, while online education just started its journey only a few decades ago and needs to be fully exploited. If teachers could appropriately integrate online teaching with offline teaching, it would amplify the effectiveness of the construction of the medical postgraduate training paradigm and exercise postgraduates' ability to think, discuss and solve problems. Online education has already developed to overcome many of the shortcomings of offline teaching and forms the basis for the creation of an environment for sharing and discussion. That is especially effective in digitally advanced areas. Online education helps to form an active virtuous circle in the cultivation of academic medical postgraduates in the small-class teaching mode. This gives full play to motivating enthusiasm for and participation in student-centered learning and to integrating teaching resources, exerting the effect of “one plus one is greater than two” and thus paving the way for a promising future for the further reform of medical education. However, we have to point out the limitations here, as the conclusions of this study are mainly based on local teaching practices in well-established network infrastructures. Less developed areas with difficulty accessing the latest digital technologies might produce more critical results.

## Author Contributions

BS conceptualized the study, searched and analyzed the literature, and wrote the draft of the manuscript.

## Funding

This work was supported by the National Natural Science Foundation of China (NSFC, 81974303 and 81772165), the National 13^th^ Five-Year Grand Program on Key Infectious Disease Control (2017ZX10202102-005-003), the Climbing the Peak (Dengfeng) Talent Training Program of Beijing Hospitals Authority (DFL20191701), the China Primary Health Care Foundation-Youan Medical Development Fund (BJYAYY-2020PY-01), the Funding for Chinese overseas talents returning to China in 2016, and the Beijing Key Laboratory for HIV/AIDS Research (BZ0089). The funders had no role in study design, data collection and analysis, decision to publish, or preparation of the manuscript.

## Conflict of Interest

The author declares that the research was conducted in the absence of any commercial or financial relationships that could be construed as a potential conflict of interest.

## Publisher's Note

All claims expressed in this article are solely those of the authors and do not necessarily represent those of their affiliated organizations, or those of the publisher, the editors and the reviewers. Any product that may be evaluated in this article, or claim that may be made by its manufacturer, is not guaranteed or endorsed by the publisher.
